# Eosinophils, Hypoxia-Inducible Factors, and Barrier Dysfunction in Functional Dyspepsia

**DOI:** 10.3389/falgy.2022.851482

**Published:** 2022-05-31

**Authors:** Suraj Hari, Grace L. Burns, Emily C. Hoedt, Simon Keely, Nicholas J. Talley

**Affiliations:** ^1^Tasmanian School of Medicine, College of Health and Medicine, University of Tasmania, Hobart, TAS, Australia; ^2^NHMRC Centre of Research Excellence in Digestive Health, University of Newcastle, Newcastle, NSW, Australia; ^3^Hunter Medical Research Institute, New Lambton Heights, NSW, Australia; ^4^School of Biomedical Sciences and Pharmacy, College of Health, Medicine and Wellbeing, The University of Newcastle, Newcastle, NSW, Australia; ^5^School of Medicine and Public Health, College of Health, Medicine and Wellbeing, The University of Newcastle, Newcastle, NSW, Australia

**Keywords:** eosinophils, hypoxia, functional dyspepsia, barrier integrity, functional gastrointestinal disorder, inflammation

## Abstract

Functional dyspepsia (FD) is a highly prevalent disorder of gut-brain interaction (DGBI), previously known as a functional gastrointestinal disorder. Characterized by early satiety, postprandial fullness, and/or epigastric pain or burning, diagnosis depends on positive symptomatology and exclusion of obvious structural diseases. A subtle inflammatory phenotype has been identified in FD patients, involving an increase in duodenal mucosal eosinophils, and imbalances in the duodenal gut microbiota. A dysregulated epithelial barrier has also been well described in FD and is thought to be a contributing factor to the low-grade duodenal inflammation observed, however the mechanisms underpinning this are poorly understood. One possible explanation is that alterations in the microbiota and increased immune cells can result in the activation of cellular stress response pathways to perpetuate epithelial barrier dysregulation. One such cellular response pathway involves the stabilization of hypoxia-inducible factors (HIF). HIF, a transcriptional protein involved in the cellular recognition and adaptation to hypoxia, has been identified as a critical component of various pathologies, from cancer to inflammatory bowel disease (IBD). While the contribution of HIF to subtle inflammation, such as that seen in FD, is unknown, HIF has been shown to have roles in regulating the inflammatory response, particularly the recruitment of eosinophils, as well as maintaining epithelial barrier structure and function. As such, we aim to review our present understanding of the involvement of eosinophils, barrier dysfunction, and the changes to the gut microbiota including the potential pathways and mechanisms of HIF in FD. A combination of PubMed searches using the Mesh terms functional dyspepsia, functional gastrointestinal disorders, disorders of gut-brain interaction, duodenal eosinophilia, barrier dysfunction, gut microbiota, gut dysbiosis, low-grade duodenal inflammation, hypoxia-inducible factors (or HIF), and/or intestinal inflammation were undertaken in the writing of this narrative review to ensure relevant literature was included. Given the findings from various sources of literature, we propose a novel hypothesis involving a potential role for HIF in the pathophysiological mechanisms underlying FD.

## Introduction

**Figure 1:**
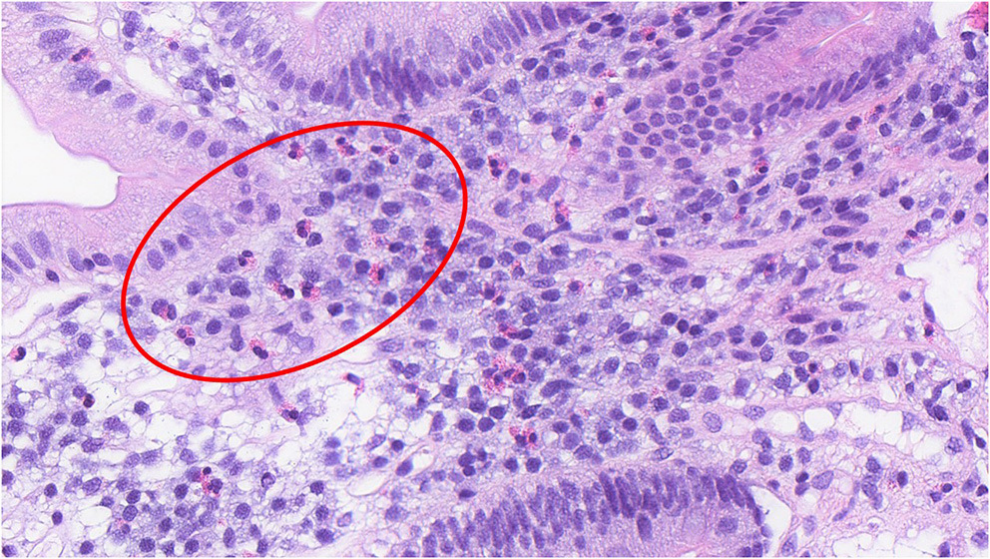
Increased duodenal eosinophils in a duodenal biopsy section of a patient with functional dyspepsia. (H&E, x40) The histological section shows the presence of duodenal eosinophils in an FD patient, noted by the bi-lobed nucleus and distinct hold of the pink, eosin stain. A group of eosinophils have been shown in the region enclosed within the red circle.

Functional dyspepsia (FD) is a chronic gastrointestinal (GI) disorder where there exists no organic explanation for the clinical presentation and patient symptom experience, thereby being labeled a disorder of gut-brain interaction (DGBI) (1). While the etiology and precise pathophysiology of FD remain poorly characterized (2), the core symptoms for diagnosis are early satiety (inability to finish a normal sized meal), postprandial fullness (often referred to as bloating by the patient), and/or epigastric pain or burning (3). These symptom profiles are categorized into FD subtypes, epigastric pain syndrome (EPS) and postprandial distress syndrome (PDS), with the aim of guiding treatment approaches, however there is significant overlap within these subtypes (4). In addition to a positive symptom profile, an exclusion of obvious structural disease on endoscopy and other routine investigations fulfills the Rome IV diagnostic criteria for FD (Table 1) (3). FD is associated with increased duodenal eosinophils in a major subgroup (5, 6), which are not specific to either EPS or PDS subtypes (6). This is illustrated histologically in Figure 1, which shows increased eosinophils in the duodenal mucosa of an FD patient. Other GI pathologies, such as coeliac disease, Crohn's disease and eosinophilic gastroenteritis can present with upper GI symptoms and duodenal eosinophilia (7) but are not frequently confused with FD.

**Table 1:** Rome IV criteria^*^ for the diagnosis of functional dyspepsia and subtypes of functional dyspepsia.

Functional Dyspepsia		Must include one or more of: • Bothersome postprandial fullness (i.e., feeling uncomfortably full after a regular sized meal) • Bothersome early satiation (i.e., unable to finish a regular sized meal) • Bothersome epigastric pain and/or burning
		AND • No evidence of any structural disease (including at upper endoscopy) that is likely to explain the symptoms
	*Postprandial Distress Syndrome (PDS)*	Must include one or both of the following at least 3 days a week: • Bothersome postprandial fullness that is severe enough to impact on usual activities • Early satiety that is severe enough to prevent finishing a regular sized meal
		AND • No evidence of any organic, systemic, or metabolic disease that may explain symptoms on routine investigations, including upper endoscopy.
	*Epigastric Pain Syndrome (EPS)*	Must include one or both of the following at least 1 day a week: • Epigastric pain and/or burning that is severe enough to impact on usual activities
		AND • No evidence of any organic, systemic, or metabolic disease that may explain symptoms on routine investigations, including upper endoscopy.

^*^All criteria listed in the Rome IV classification must be fulfilled for the last 3 months with symptom onset at least 6 months prior to diagnosis being made.

The reported prevalence of FD varies, from 7.2% (8) to approximately 16% (9), and the heterogeneity in prevalence data is likely due to variation based on country and the definition used for FD diagnosis. In addition, FD has a serious impact on patient quality of life (1, 10). Studies have also demonstrated FD patients quality of life impairment is similar to patients with mild heart failure (11, 12). Studies have shown a notable increase in work absences and annual medical costs in FD patients, creating an estimated $8,544 USD worth of losses in productivity compared to $3,039 USD in non-FD individuals (13, 14). Taken together, it is clear that FD is a prevalent condition that poses significant costs to not only a patient's quality of life, but healthcare and economic systems as well.

The underlying pathophysiology of FD is not comprehensively understood, largely owing to heterogeneity in symptom profiles (9). With over 34% of FD patients suffering from a psychiatric condition (15), the involvement of a complex, bidirectional relationship between the gastroduodenal region and the brain (the gut-brain axis) in driving patient symptomatology and psycho-social pathology is becoming widely accepted (16). As such, recent nomenclature has shifted toward referring to FD as a disorder of gut-brain interaction (DGBI) to better reflect the biopsychosocial nature of the condition and the current scientific understanding (1). Of the various DGBIs, FD is thought to be one of the most common (17).

Evidence has emerged of a subtle inflammatory phenotype in FD patients (18), characterized by increased peripheral gut-homing T cells, duodenal eosinophils and increased barrier permeability, separating this type of condition from organic pathologies which tend to present with more obvious biochemical and structural signs. The precise involvement of the gut-brain axis in potentiating this phenotype is not clear, with 50% of functional gastrointestinal disease (FGID) cases commencing with psychological distress prior to GI symptoms, whilst the remaining 50% of cases present with gut dysfunction first prior to developing psychosocial pathology (1). While specific mechanisms are yet to be identified, a range of physiological abnormalities have been implicated as contributors to symptom generation and the inflammatory phenotype including visceral hypersensitivity (19, 20), disturbances in gastric motility (19, 20), post-infectious gastroenteritis (21), changes in bile acid composition (22), increased intestinal permeability (23, 24), immune dysfunction (9, 18), and alterations to the gut microbiota (25). The clinical subtypes of FD are not established to have different pathophysiological mechanisms (26).

### Immune Activation, the Gut Microbiota, and Duodenal Barrier Dysfunction

Given that a loss in mucosal integrity is a central feature in the low-grade duodenal inflammation associated with FD (27), dysfunction of the intestinal immune system and the gut microbiota may have a role in potentiating cellular stress and maladaptive changes to the duodenal barrier (9). Whilst the precise role of eosinophils in FD pathology is yet to be comprehensively understood (28), eosinophils are both effector cells of the Th-2 pathway (29) and regulatory cells of the Th-17 response (30). Taken together with evidence of an increased presence of gut-homing lymphocytes (31) and mucosal Th-17 cells (32) suggesting an adaptive immune response in FD, there exists a possible pathway through which eosinophils may participate in the FD inflammatory phenotype. In fact, this is reinforced by a review which posited an interplay between Th2 and Th17 as a central feature of the inflammatory profile in FD (33). Interestingly, a greater level of eosinophil, macrophage, and intraepithelial lymphocyte infiltration has been observed within the duodenal mucosa of patients with FD following infectious gastroenteritis (34). In fact, an earlier retrospective study by Tack et al. showed a greater symptom prevalence in patients with a history of suggestive post-infectious dyspepsia as opposed to FD patients with an unspecified onset, suggesting that infection serves as an important trigger for the onset and prevalence of dyspeptic symptoms and inappropriate immune activation in FD (35). Further, SARSCoV2 has been associated with an increased risk of postinfectious functional GI disorders including FD (36).

In a more recent cross-sectional study involving a subset of ethnically diverse adults, Järbrink-Sehgal et al. reported an increased degranulation of duodenal eosinophils in FD (37). Activated eosinophils may potentiate epithelial barrier dysfunction in FD through stimulating the release of proinflammatory mediators, of which tumor necrosis factor (TNF), and interleukin-1β (IL-1β) have been shown to be significantly raised in FD patients (31, 38). Whilst not extensively studied in FD, age-related intestinal dysbiosis, amongst other factors such as physical inactivity and diet, has been demonstrated as an important trigger for dysregulation of innate immunity, represented by consistently elevated levels of pro-inflammatory mediators, such as TNF and IL-1β (39). However, the exact impact of age in FD is uncertain. Proinflammatory cytokines can initiate and contribute to cellular stress pathways and epithelial tissue damage, thereby also potentially compromising the integrity of the duodenal barrier (40). In fact, Komori et al. recently reported an association between IL-1β levels and barrier permeability in FD patients, highlighting the effects that pro-inflammatory cytokines in FD may have in potentiating cellular stress and low-grade duodenal inflammation (24). Additionally, intestinal epithelial cell lines exposed to Major Basic Protein (MBP), an eosinophil degranulation protein, exhibited a loss of barrier function, thence further linking eosinophils as contributors to epithelial stress and dysfunction (41).

Alongside duodenal eosinophilia, alterations to the small intestinal gut microbiota serve as a potential contributing factor to the low-grade duodenal inflammation (9). As diet is intricately linked with the microbiota it is likely to be an important contributor to immune and microbial interactions in FD and has been reviewed previously (42). In fact, FD has been shown to be more prevalent in populations with increased body fat and obesity (43–45). Physiologically, the duodenal microbiota plays a crucial role in supporting host digestive function within the small intestine through the fermentation of foods and release of digestive enzymes (42), such as bile salt hydrolase (46, 47). However, in environments where the gut microbiota is altered, such as FD, a state of microbial “dysbiosis” can be a driving force in GI symptom generation and the pathology itself (25, 42). Alterations in the microbiota, and thus their functional repertoire, can induce changes in both the short-chain fatty acid (SCFA) profile, primary metabolites of fermentation, as well as the bile acid pool (48, 49). Changes in the bile acid composition can consequently perpetuate further changes in gut microbial diversity (47) and can drive epithelial stress and damage downstream (22). A hypothesis that may explain this occurrence is that reduction in bile acid concentrations may lead to an overgrowth of pro-inflammatory bacteria, culminating in further cellular stress responses, epithelial barrier dysfunction and low-grade inflammation (48, 50, 51).

The exact pathological interactions between the gut microbiota, duodenal eosinophilia, and barrier dysfunction in FD remains enigmatic. The activation of cellular stress response pathways due to microbial alterations and inflammatory immune responses may trigger imbalances in gut homeostasis and facilitate the loss of mucosal integrity, ultimately driving eosinophil recruitment in response (52), thus further propagating the immune response and symptom generation in FD (9). Hypoxia-Inducible Factors (HIF) are part of a cellular pathway that is associated with the above features of disease and, therefore, may have a potential role within FD pathophysiology. As such, this paper seeks to propose a potential role for HIF in the pathophysiology of FD, utilizing evidence from previous studies in organic pathologies, such as eosinophilic oesophagitis (EoE).

### The Hypoxia-Inducible Factors

**Figure 2:**
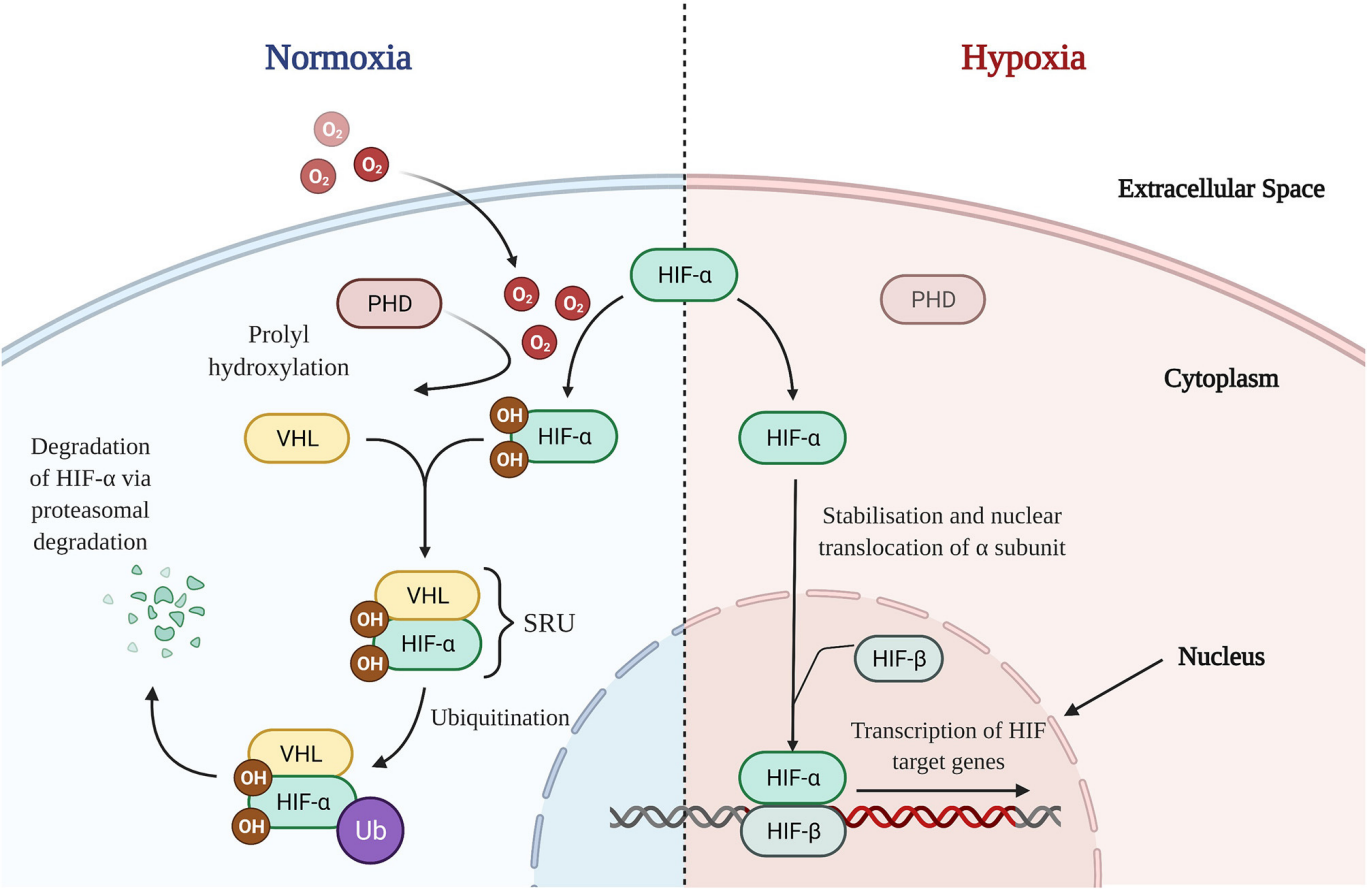
HIF Signaling Pathways in Normoxic and Hypoxic States. Normoxia: In the presence of oxygen, PHD enzymes are able to hydroxylate proline residues on the HIF-α subunit's oxygen dependent domain allowing for recognition and binding with a VHL containing protein complex (pVHL). Forming the SRU, the pVHL alongside other enzymes ubiquitinates the HIF-α subunit, marking it for proteasomal degradation and thence destabilizing it. Hypoxia: In the absence of oxygen, PHD enzymes are unable to successfully hydroxylate the oxygen dependent domain of HIF-α. This results in stabilization and a consequent accumulation of HIF-α subunits in the cytoplasm. After which, HIF-α translocates to the nucleus, heterodimerises with nuclear HIF-β, binds to the promotor region and commences the transcriptional regulation of downstream HIF target genes. *O2, Oxygen; OH, hydroxyl group; PHD, prolyl hydroxylase domain; HIF, hypoxia-inducible factor; VHL, von-Hippel-Lindau; SRU, substrate recognition unit; Ub, ubiquitin*.

HIFs are heterogenous dimers consisting of an oxygen dependent α subunit and a constitutively expressed β subunit (53). Three subsets of α units have been identified in humans: HIF-1α, HIF-2α, and HIF-3α. However, given the paucity in literature regarding the role of HIF-3α in the intestine, HIF-1α and HIF-2α will form the focus of this review (54). HIF-α proteins contain proline residues which combine to form the oxygen dependent domain (ODD), a portion of the α subunit that is hydroxylated by prolyl hydroxylase domain (PHD) enzymes, in the presence of oxygen (55) (Figure 2). In HIF-1α these pertain to proline residues 402 and 562, whilst in HIF-2α it consists of residues 405 and 531, respectively (56). Hydroxylation of the proline residues enables the HIF-α subunit to be recognized by a protein complex which also includes the von-Hippel-Lindau protein (pVHL), thereby forming the substrate recognition unit (SRU) (53) (Figure 2). Combined with other enzymes, the SRU then ubiquitinates HIF-α proteins for proteasomal degradation (53) (Figure 2). However, in states of hypoxia, oxygen deprivation inhibits PHD enzymes from hydroxylation of the ODD (57) (Figure 2). This consequently leads to the cytoplasmic accumulation of HIF-α subunits, termed HIF-α stabilization, after which nuclear translocation and heterodimerisation of the α subunit takes place facilitating binding to HIF-β subunits (Figure 2). The nuclear HIF-α/HIF-β complex binds to the promoter regions of target genes, enabling active transcription and expression of HIF downstream targets (Figure 2), such as Vascular Endothelial Growth Factor (VEGF) and erythropoietin (53). Potent eosinophil recruiting chemokines, such as eotaxins, as well as tight junction proteins have been shown to also be downstream targets of HIF, thereby illustrating a role for HIF in tissue eosinophilia (58) and the maintenance of the epithelial barrier structure and function (54).

Whilst hypoxia dependent mechanisms of HIF regulation have been the most studied, a growing body of evidence highlights a range of non-hypoxic pathways that have shown capacity toward activation and stabilization of HIFs (59). Normoxic stabilization of HIF has been shown to play an important role in the host immune selection and adaptation in cancer, thereby contributing to the progression of disease (60). Under normoxic conditions, Frede et al. reported a functional relationship between bacterial lipopolysaccharide (LPS) and the induction of HIF-1α mRNA expression and protein accumulation in human monocytic cell lines (61). Additionally, Peyssonnaux et al. also demonstrated that the non-hypoxic related stimulation of mice that had undergone conditional gene targeting of HIF-1α by an LPS challenge in the context of sepsis, significantly promoted HIF-1α mediated production of a host of inflammatory cytokines, including TNF (62). Interestingly, inflammatory cytokines have also been shown to play a part in the normoxic transcriptional regulation of HIF. For example, alongside LPS, TNF, and IL-1β were also shown to contribute to increases in HIF-1α mRNA in fibroblast cultures under normoxic conditions (63). Other work has also shown similar associations between TNF and increased HIF-1α mRNA and protein expression in human embryonic kidney cell cultures that had been treated with TNF for several hours (64). The expression of HIF-1α in these cell cultures also correlated with increased expression of HIF downstream targets, suggesting a transcriptionally active HIF response following inflammatory cytokine stimulation (64). Whilst not only evidencing an important role for HIF in the production and induction of inflammation in disease, such findings also establish capacity for microbial factors and inflammatory cytokines, such as TNF and IL-1β alike, in the oxygen-independent and normoxic stabilization of HIF transcriptional pathways.

Generally, HIF-1α and HIF-2α have been understood as two sides of a coin in terms of their transcriptional effects in intestinal inflammation (65). Whilst HIF-1α is thought to induce a barrier-protective role through the up-regulation of tight-junctional proteins and anti-microbial properties, HIF-2α stabilization increases the expression of pro-inflammatory cytokines and chemokines (65). In pathological states, a dysregulation of both HIF-1α and HIF-2α has been associated with pro-inflammatory states and/or maladaptive changes, such as transcriptional changes in tight junction expression and compromises in barrier integrity (66, 67). Although not investigated in DGBIs, given the associations between HIF and eosinophil recruitment in other studies (58, 68), there may exist a role for HIF in mediating duodenal eosinophilia and barrier dysfunction in FD which warrants further investigation.

### Eosinophilia, FD, and HIFs

In addition to increases in duodenal eosinophil numbers (6, 69), increased degranulation of these cells has also been demonstrated, suggesting the eosinophils are active in FD (5). Further, an association between duodenal eosinophilia in FD and symptoms of early satiety, postprandial fullness, and abdominal pain has also been reported (70), linking the immune cells directly to symptom burden. A potential explanation for this is that FD patients demonstrate a greater number of eosinophils in close proximity to submucosal plexus neurons which correlated with impaired neuronal function (71). Given that the submucosal plexus is involved in the mediation of local gut contraction and reflex responses (72, 73), it suggests that abnormal stimulation of submucosal plexus neurons by eosinophil inflammatory mediators may be a contributor to the manifestations of FD, such as disturbances in gastric motility. Similar findings have been made in irritable bowel syndrome (IBS), where mast cells in close proximity to intestinal nerves correlated with both the severity and frequency of abdominal pain and discomfort in IBS patients (74). Further, recent findings by Wauters et al. suggested that the effectiveness of proton pump inhibitors (PPIs) in symptom management may be attributable to suppression of eosinophils (75). As such, there exists evidence that posits a role for eosinophils in perpetuating low-grade inflammation and gut-brain dysfunction, as well as symptom generation in FD.

Activation of HIF stabilization pathways have demonstrated capacity to regulate the recruitment of eosinophils. For example, studies investigating cobalt-induced airway inflammation report pronounced eosinophilic infiltration in HIF-1α deficient mice (76), and deletion of HIF-2α results in prolonged eosinophilic infiltration in animal models (77), suggesting HIF may act as a regulatory checkpoint for eosinophilic inflammation. TNF and IL-1β are both pro-inflammatory cytokines that have been identified as being significantly raised in FD patients (31, 38). Studies investigating airway inflammation have shown that both TNF and IL-1β also serve as contributors to eosinophil recruitment (78, 79). Relevant to the GI tract, the role of TNF in mediating eosinophil recruitment is also specifically highlighted in a study that reported a significant inhibition in eosinophil chemotactic ability in ulcerative colitis patients whose perfusion fluids had been treated with anti-TNF (80). Therefore, it may also be possible for TNF and IL-1β signaling to also contribute to eosinophil recruitment in FD. HIF-1α has generated intense interest as a therapeutic target for inflammatory bowel disease, stemming from the initial observations that HIF-1α knockout animals were protected against chemically-induced colitis, while VHL knockout animals (that exhibit constitutive HIF-1α stabilization) were protected against colitis (81). This has led to the approach of pharmacologically stabilizing intestinal HIF-1α to protect against colitis (82–84) and this has progressed to clinical trials (85). Mechanistic studies suggest that the epithelium is the key component of this therapeutic efficacy (86, 87) although HIF-1α also controls dendritic cell activation of protective regulatory T cells suggesting that HIF-1α is important for regulation of underlying lamina propria mononuclear cells (88). Conversely, overexpression of HIF-2α in intestinal epithelial cells lead to spontaneous DSS colitis in mice coinciding with an increased expression of TNF, IL-1β and IL-6, whilst deletion of HIF-2α in mice with DSS-induced colitis has a protective effect (89). Whilst highlighting the contrasting roles of HIF-1α and HIF-2α, these findings also indicate the importance in maintaining balance between HIF-1α and HIF-2α in regards to shaping the response that occurs to an inflammatory or pathological stressor, alongside reinforcing how a dysregulation in this balance may trigger maladaptive changes (65, 90). The existing evidence suggests a role for HIF-1/HIF-2 balance in the downstream regulation of TNF and IL-1β, pro-inflammatory cytokines involved with the mobilization and recruitment of eosinophils. Although mouse models of colitis are not directly comparable to human presentations of functional disease, given the TNF and IL-1β inflammatory profile in a subset of FD patients, a dysregulation in HIF transcriptional pathways, specifically in the form of a downregulation in HIF-1α, may provide a possible explanation for eosinophil recruitment and barrier dysfunction in FD pathophysiology.

### Barrier Dysfunction, FD, and HIFs

**Figure 3:**
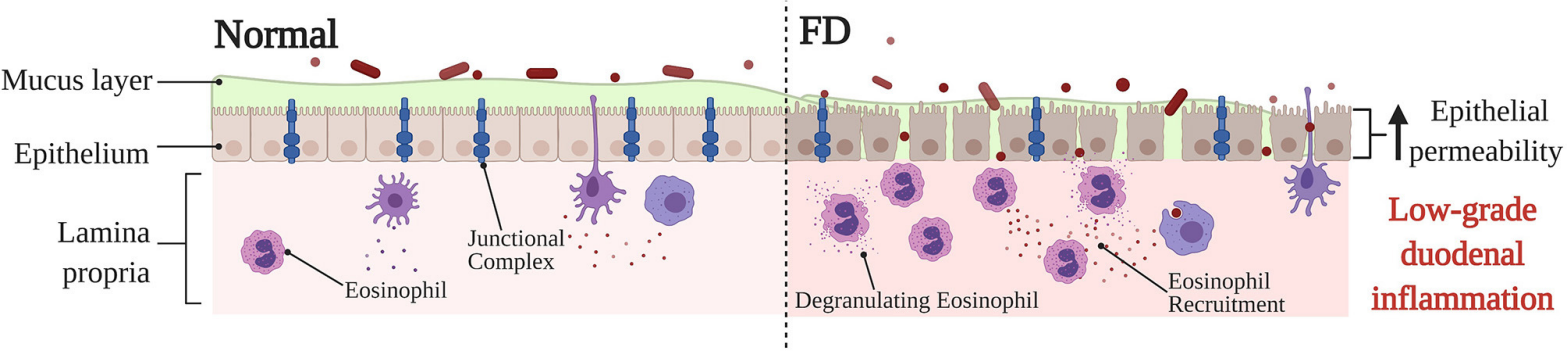
Barrier dysfunction in FD compared to normal, baseline conditions. Under physiological conditions, the duodenal barrier consists of a mucous layer, epithelium and underlying lamina propria. The integrity of the epithelial barrier is maintained via a network of junctional complexes, made up of tight junction proteins, such as zonula occludens-1, occludin and claudin-1. Due to the being under constant physiological stress the intestinal epithelium, the lamina propria typically contains immune cells to maintain and protect the barrier. In FD, a dysfunction of the barrier has been described specifically in relation to a loss in mucosal integrity and increased permeability. The expression of adhesion proteins is altered which lends to the severity of the low-grade duodenal inflammation observed in FD. Furthermore, the recruitment and activation of eosinophils in FD also takes place, which may perpetuate tissue damage and barrier dysfunction thereby also adding to the low-grade FD barrier inflammation. The exact reason for the barrier dysfunction, and also whether the changes in barrier are causative, or rather a consequence of FD pathology is still poorly understood. *FD, Functional Dyspepsia*.

The intestinal barrier serves as an important semipermeable interface between the external environment and internal body systems, specifically allowing the uptake of essential nutrients and immune surveillance whilst also simultaneously restricting the passage of pathogenic microorganisms and molecules (91). Part of the many systems at play ensuring the integrity of the epithelial barrier is maintained are a network of protein structures that form the cell junction which links cells within the epithelium (91, 92) (Figure 3). These junctional complexes are comprised of a combination of three different components: tight junctions, adherens junctions, and desmosomes (92). Tight junctions play a crucial role in regulating paracellular transport between cells via two distinct pathways; the pore and leak pathway (93). Where the pore pathway facilitates passage of small ions and uncharged molecules, the leak pathway allows for the passage of larger ions and macromolecules irrespective of charge (93). A dysregulation of tight junctions has been recognized as a potentiating factor to changes in intestinal permeability, intestinal barrier loss and disease (93). Dysfunction of the duodenal epithelial barrier has been described in FD as losses in mucosal integrity and increases in barrier permeability (19, 23) (Figure 3). Komori et al. observed decreases in expression of zonula occludens-1 (ZO-1), a tight junction protein found within the intestinal barrier, in FD patients relative to a symptomatic control group with abdominal pain (24). This finding is also supported by Vanheel et al. who also demonstrated a significant association between impaired mucosal integrity and the abnormal gene expression of proteins associated with junctional complexes, namely occludin, β-catenin, and desmosomal proteins (23). The increase in epithelial permeability was reported to be significantly associated with the severity of low-grade duodenal inflammation and protein expression of occludin was correlated with duodenal eosinophil counts (23), highlighting the importance of the duodenal barrier in potentiating the FD inflammatory phenotype as well as the role eosinophils may play in contributing to the dysfunction of the duodenal barrier. A more recent study has also revealed a significantly reduced duodenal expression of claudin-1, a tight junction protein, in FD patients against healthy controls, which remained statistically significant after adjustment for potential confounding factors, including age and sex (94). Whilst these findings, overall, fail to clarify whether the loss in barrier integrity is causative, or rather, a consequent manifestation of the FD pathological process (95), it does position the duodenal barrier as an important factor in FD pathophysiology. Given that the findings show alteration in expression of proteins that make up the cell junction, the nature of barrier dysfunction in FD likely involves the paracellular pathway. In fact, an increased paracellular permeability has already been reported in organic intestinal pathologies such as Crohn's disease (96). While a number of studies highlight altered permeability as a feature of FD, the identification of precise cellular pathways is a limitation of the literature given that most of the applied methodologies only assess singular aspects of cellular permeability (97). As such, further investigation of the precise cellular pathways that underpin barrier dysfunction may have the potential to better the current understanding of the FD pathological process.

Emerging research has shown a strong relationship between HIF pathways and the downstream expression of tight junction proteins. In a 2015 study involving a HIF-1α chromatin immunoprecipitation analysis in intestinal epithelial cell cultures, Saeedi et al. identified claudin-1 as a prominent HIF downstream transcriptional target (98). Interestingly, significant repression of both HIF-1α and claudin-1 has been reported in active EoE (66). When translated to a transgenic mouse model of EoE which overexpressed HIF-1α, the group noted an attenuation of inflammation which correlated with the restoration of esophageal claudin-1 expression (66). These findings evidence the necessity of HIF-1α signaling in barrier maintenance and the niche role of claudin-1 in the barrier. Another rat model study observed that chronic increases in HIF-2α lead to a higher turnover in the expression of the tight junction proteins ZO-1 and occludin (99), both of which have also been reported as being decreased in FD at both the protein and gene expression level (23, 24). Therefore, there may be a transcriptional influence, such as HIF pathways, underpinning the decreased expression of epithelial barrier proteins in FD.

The associations between HIF and the transcriptional regulation of cell junctional complexes presents a possible rationale for dysregulated HIF cellular responses as an underpinning factor influencing the duodenal barrier dysfunction in FD. It is however acknowledged that EoE represents a subset of organic pathologies that present with pronounced barrier dysfunction and inflammation (66), and may not be directly comparable to the subtle inflammation characteristic of FD (18). Therefore, it is likely that if a dysregulation of the HIF pathway is present in FD, the nature of the dysregulation may be unique to functional conditions and more nuanced in comparison to pathologies such as EoE.

### The Gut Microbiota, FD, and HIFs

The gut microbiota plays a significant role in the homeostatic maintenance of the intestinal barrier (100, 101), the digestion, metabolism and absorption of vital nutrients (102), and the intestinal immune response (25). As such, alterations in the microbiota of the duodenum have been suggested to play a role in the pathophysiology of DGBIs, including FD (42, 49). Studies have reported both taxonomic and microbial load differences in the duodenum of FD patients. In a 2016 prospective cohort study assessing the microbiota profiles of gastric fluid from 44 FD patients against 44 healthy controls reported a higher relative abundance of *Prevotella* (Bacteroidetes) in FD (103). While microbiota studies can be conflicting, largely due to differences in sequencing targets and analysis, there appears to be a growing consensus that report increases in the relative abundance of *Streptococcus* species in FD (104, 105). Furthermore, Fukui et al. also reported a positive correlation between *Streptococcus* relative abundance and FD symptoms (105).

Increased microbial load has also been reported in FD duodenal samples. Shah et al. compared the loads of DGBI patients (FD and irritable bowel syndrome), IBD patients, and asymptomatic controls found that FD patients demonstrated an increased duodenal microbial load relative to asymptomatic controls (106). Further to this, Zhong et al. reported a positive correlation between microbial load and the intensity of meal-related FD symptoms, whilst also finding a negative correlation between microbial load and patient quality of life (107). Interestingly, high-glucose and high- fructose diets in experimental mice have been associated with dysbiosis as well as impairments of the intestinal barrier; mice fed such diets exhibited a decreased expression of tight junctions ZO-1 and occludin, as well as an increase in levels of TNF and IL-1β; all of which have also been reported to be altered in FD (108). Despite paucity and heterogeneity existing within the literature, it is evident that gut microbiota imbalances likely have a role to play in driving FD.

The therapeutic potential of probiotic supplementation in restoring microbial niches and reducing GI symptoms in FD has shown recent promise. In a 2017 study, Igarashi et al. not only reported alterations in the taxonomic profiles of FD patient gastric fluid, but also noted a “positive” shift in the gastric fluid microbial composition after probiotic treatment to that which was observed in healthy control volunteers (109). This study used a probiotic product containing *Lactobacillus* spp., a common probiotic genus. *Lactobacilli* are commonly referred to as lactic acid bacteria, capable of producing SCFAs lactate, acetate and butyrate (110). The viability of probiotic administration for FD has also been reported by Drago et al. here authors observed a significant reduction in symptom prevalence amongst PDS patients treated with probiotics alone (111). Furthermore, a recent placebo-controlled pilot trial also showed evidence of potentially beneficial immune and microbial changes in FD patients administered probiotic treatment against those on placebo (112). Whilst further research is still required, evidence does suggest that probiotics or their metabolic by-products may serve as a viable therapeutic option in FD treatment and symptom management.

A part of the many functions of the gut microbiota involves the fermentation of indigestible polysaccharides, such as dietary fiber, producing SCFAs such as butyrate, an “anti-inflammatory” metabolite (113). SCFAs play an important role in regulating intestinal structure and inflammation, therefore, alterations in microbial niches responsible for the production and maintenance of “healthy” levels of SCFAs may lead to gut-barrier dysfunction and low-grade inflammation (114). Although the SCFA profile of the FD duodenum still remains to be characterized, changes have been observed in irritable bowel syndrome, an DGBI that shares overlap with FD (115). A meta-analysis found that butyrate levels were decreased in constipation dominated IBS whilst being raised in diarrhea predominant IBS (116). Animal work examining the effects of SCFAs in GI motility concluded that increased transit rate in the mouse IBS model group is associated with a chronically elevated SCFA profile (117). Further evidence also demonstrates reduced contractile responses with increasing concentrations of butyrate (118). Not only do these findings allude to an altered SCFA profile in DGBI, but also support a possible role for SCFAs in driving gut motility disturbances in FD (119).

The metabolization of SCFAs through β-oxidation within intestinal epithelial cells is an oxygen-intensive process, which under physiological conditions has been shown to influence the stabilization of HIF (51, 120). Kelly et al. reported a significant correlation between bacteria-derived butyrate and increased stabilization of HIF in mice, and additionally reported that antibiotic mediated depletion of microbiota resulted in reduced butyrate and HIF expression, which was later corrected by butyrate supplementation (121). Given the evidence to suggest that probiotics are able to shift the microbial composition and in turn restore production of SCFAs in the intestine (122), there is the possibility that the therapeutic effectiveness of probiotic supplementation in FD is due to a probiotic mediated resolution of the gut microbiota, SCFAs, and in turn a dysregulated HIF stabilization pathway.

## Discussion: Hypothetical Role of HIFS in the Pathophysiology of FD

Despite being a highly prevalent DGBI, the etiology and pathophysiology of FD is not comprehensively understood. Hallmarked by experiences of early satiety, postprandial fullness, and/or epigastric pain or burning, FD has been shown to have a significant impact on patient quality of life (123). A subtle, low-grade inflammatory phenotype involving an increase in tissue eosinophils, dysfunction of the duodenal barrier, and alterations in the gut microbiota has been identified in patients and are thought to be contributing factors to the pathophysiology (18, 124). Duodenal eosinophilia and gut dysbiosis are associated with tissue damage and have a role in potentiating the loss in barrier integrity, and in turn low-grade mucosal inflammation (1, 9).

The HIF transcriptional pathway is an example of a cellular pathway that is associated with the above features of disease. Most notably, the existing literature points toward a role for HIF in regulating the proinflammatory response, including the recruitment of eosinophils, as well as in the maintenance of the epithelial barrier (58, 65). There are also findings that establish a functional link between the gut microbiota and HIF, specifically the microbiota mediated stabilization of HIF via SCFA metabolism (121).

**Figure 4:**
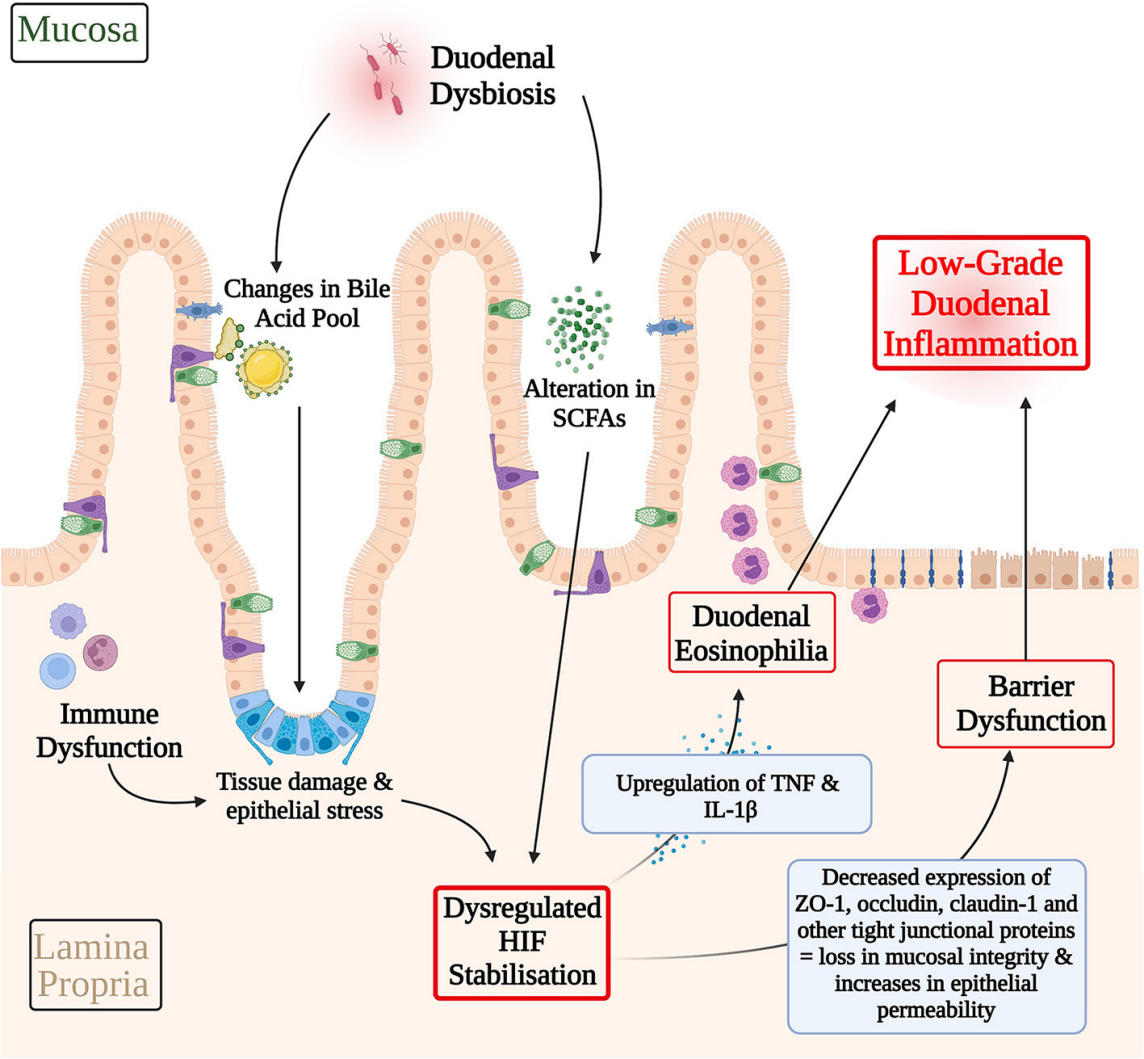
Hypothetical involvement of HIF in the pathophysiology of functional dyspepsia. A dysbiotic gut microbiota may lead to alterations in the SCFA profile and bile acid pool and therefore the inappropriate activation of cellular stress response pathways, such as HIF. Immune dysfunction may serve as another contributing factor to cellular stress response pathways via pro-inflammatory cytokine production and consequent epithelial tissue damage. A dysregulated HIF transcriptional pathway may have downstream effects on the expression of HIF transcriptional targets that mediate eosinophil recruitment and barrier structure in FD. For example, HIF mediated upregulation of TNF and IL-1β, which have been shown to be raised in FD inflammation, may contribute to FD duodenal eosinophilia. Similarly, decreases in the expression of ZO-1, occludin, and claudin-1 have been described in FD. Given the role of HIF in maintaining the integrity of the epithelial barrier, HIF dysregulation presents a possible pathway through which the differences in expression of these cell adhesion proteins and in turn barrier dysfunction in FD may be explained. Both, duodenal eosinophilia and losses in mucosal integrity serve as key contributing factors to the low-grade duodenal inflammation seen in FD. In this way, HIF dysregulation may have a hypothetical role to play in driving the pathophysiology of FD. *SCFAs, Short-chain fatty acids; HIF, Hypoxia-inducible factor; TNF, Tumor Necrosis Factor; IL-1*β*, Interleukin-1*β*; ZO-1, Zonula occludens-1*.

In culmination, it is possible that an imbalanced gut microbiota may lead to alterations in the SCFA profile (125) and bile acid pool (48), and thereby dysregulating cellular stress response pathways in FD, such as HIF. Immune dysfunction may also potentiate cellular stress through pro-inflammatory cytokine production and consequent epithelial tissue damage (9). These pathological alterations in HIF levels may have effects in terms of the transcription and expression of HIF downstream targets. TNF and IL-1β are examples of HIF responsive pro-inflammatory cytokines that are involved with eosinophil recruitment, which have also been found to be significantly raised in FD (31, 38, 78, 79, 89, 126). Additionally, a dysregulated HIF stabilization pathway may also manifest in the altered expression of junctional complexes, therefore compromising barrier integrity (66). This has been reported in studies of EoE, where a dysregulation in HIF lead to a consequent reduction in claudin-1 levels therefore perpetuating the barrier dysfunction (66). Specifically, to FD, ZO-1, occludin, and claudin-1 are examples of tight junction proteins that have been shown to have a decreased expression that are also downstream targets of HIF (23, 24, 94, 99). In this way, dysregulated HIF pathways may also underpin the expression of junctional complexes and therefore barrier dysfunction in FD. The resulting loss in duodenal integrity may allow for secondary luminal antigens and recruitment of duodenal eosinophils, therefore further propagating immune dysfunction, gut dysbiosis, and the low-grade inflammatory phenotype in FD (9). This hypothesized pathological pathway is summarized in Figure 4.

Experimental therapeutics in animal models have demonstrated a barrier protective role for HIF stabilizing drugs (82–84, 86, 87, 127, 128). Contingent upon further research confirming a role for HIF in FD pathology, there exists the therapeutic potential for HIF stabilizing drugs to augment the current therapeutic options available in the FD treatment and symptom management, and these should be considered in future investigations.

The pathophysiology of FD remains enigmatic and poorly understood. Whilst multiple contributors to the disease process have been identified, the precise mechanism of disease and the complex interactions that take place during this process remain the subject of further research. In this paper, we have proposed the HIF transcriptional pathway as another possible contributor to the FD disease process, in the hope that this may prompt new avenues in FD research. It is, however, acknowledged that findings from organic pathologies cannot be directly translated to a functional disease, which is only further complicated by the paucity in investigations of HIF pathways in disorders of gut-brain interactions. Given the existing evidence, we believe that there may exist the potential for HIF involvement in driving the pathophysiology of FD by establishing links between FD eosinophilia, barrier dysfunction, and gut dysbiosis. Further experimental research is required to validate our novel hypothesis and conclusively characterize if there is a role for HIF in FD pathophysiology.

## Author Contributions

SH drafted the manuscript under the supervision of GB, EH, SK and NT. GB, EH, SK, and NT provided technical support and expertise in the writing of this manuscript. All authors made edits and corrections, reviewed, and approved the final version. All figures, excluding Figure 1, were prepared by SH using BioRender.com under the supervision and guidance of GB, EH, SK, and NT. The annotation contained in Figure 1 was undertaken by SH. All authors contributed to the article and approved the submitted version.

## Funding

We acknowledge the support of funding from the National Health and Medical Research Council (NHMRC) for the Center for Research Excellence in Digestive Health and NHMRC Ideas grant.

## Conflict of Interest

SH declares no conflicts of interest. GB declares no conflicts of interest. EH declares no conflicts of interest. SK declares grant/research support from Cancer Institute NSW (Career Development Fellowship), National Health and Medical Research Council (Center of Research Excellence, Project and Ideas Grant), Viscera Labs, Gossamer Bio, Anatara Lifesciences, Microba (Funded research and consultancy). NT reports, non-financial support from HVN National Science Challenge NZ, personal fees from Aviro Health (Digestive health) (2019), Anatara Life Sciences, Brisbane (2019), Allakos (gastric eosinophilic disease) (2021), Bayer (IBS) (2020), Danone (Probiotic) (2018), Planet Innovation (Gas capsule IBS) (2020), Takeda, Japan (gastroparesis) (2019), twoXAR (2019) (IBS drugs), Viscera Labs, (USA 2021) (IBS-diarrhea), Dr Falk Pharma (2020) (EoE), Censa, Wellesley MA USA (2019) (Diabetic gastroparesis), Cadila PharmIncaceuticals (CME) (2019), Progenity Inc. San Diego, (USA 2019) (Intestinal capsule), Sanofi-aventis, Sydney (2019) (Probiotic), Glutagen (2020) (Celiac disease), ARENA Pharmaceuticals (2019) (Abdominal pain), IsoThrive (2021) (esophageal microbiome), BluMaiden (2021), Rose Pharma (2021), Intrinsic Medicine (2021), Comvita Mānuka Honey (2021) outside the submitted work; In addition, NT has a patent Nepean Dyspepsia Index (NDI) 1998, Biomarkers of IBS licensed, a patent Licensing Questionnaires Talley Bowel Disease Questionnaire licensed to Mayo/Talley, a patent Nestec European Patent licensed, and a patent Singapore Provisional Patent “Microbiota Modulation Of BDNF Tissue Repair Pathway” issued, “Diagnostic marker for functional gastrointestinal disorders” Australian Provisional Patent Application 2021901692. Committees: OzSage, Australian Medical Council (AMC) [Council Member]; Australian Telehealth Integration Programme; MBS Review Taskforce; NHMRC Principal Committee (Research Committee) Asia Pacific Association of Medical Journal Editors. Boards: GESA Board Member, Sax Institute, Committees of the Presidents of Medical Colleges. Community group: Advisory Board, IFFGD (International Foundation for Functional GI Disorders), AusEE. Miscellaneous: Avant Foundation (judging of research grants). Editorial: Medical Journal of Australia (Editor in Chief), Up to Date (Section Editor), Precision and Future Medicine, Sungkyunkwan University School of Medicine, South Korea, Med (Journal of Cell Press). NT is supported by funding from the National Health and Medical Research Council (NHMRC) to the Center for Research Excellence in Digestive Health and he holds an NHMRC Investigator grant.

## Publisher's Note

All claims expressed in this article are solely those of the authors and do not necessarily represent those of their affiliated organizations, or those of the publisher, the editors and the reviewers. Any product that may be evaluated in this article, or claim that may be made by its manufacturer, is not guaranteed or endorsed by the publisher.
